# Personalized assent for pediatric biobanks

**DOI:** 10.1186/s12910-016-0142-0

**Published:** 2016-10-12

**Authors:** Noor A. A. Giesbertz, Karen Melham, Jane Kaye, Johannes J. M. van Delden, Annelien L. Bredenoord

**Affiliations:** 1 Department of Medical Humanities, Division Julius Center, University Medical Center Utrecht, Office Stratenum 6.131, P.O. Box 85500, 3508 GA Utrecht, The Netherlands; 2 Department of Genetics, Division Biomedical Genetics, University Medical Center Utrecht, KC.04.084.2, P.O. Box 85090, 3508 AB Utrecht, The Netherlands; 3 Clinical Trials & Research Governance (CTRG) University of Oxford Joint Research Office, Block 60, Churchill Hospital, Headington, Oxford, OX3 7LE United Kingdom; 4 Nuffield Department of Population Health, HeLEX-Centre for Health, Law and Emerging Technologies at Oxford, University of Oxford, Ewert House, Ewert Place, Banbury Road, Summertown, Oxford, OX2 7DD UK

**Keywords:** Research ethics, Pediatric biobank, Assent, Consent, Children, Biological samples

## Abstract

Pediatric biobanking is considered important for generating biomedical knowledge and improving (pediatric) health care. However, the inclusion of children’s samples in biobanks involves specific ethical issues. One of the main concerns is how to appropriately engage children in the consent procedure. We suggest that children should be involved through a personalized assent procedure, which means that both the content and the process of assent are adjusted to the individual child. In this paper we provide guidance on how to put personalized assent into pediatric biobanking practice and consider both the content and process of personalized assent. In the discussion we argue that the assent procedure itself is formative. Investing in the procedure should be a requirement for pediatric biobank research. Although personalized assent will require certain efforts, the pediatric (biobank) community must be aware of its importance. The investment and trust earned can result in ongoing engagement, important longitudinal information, and stability in/for the research infrastructure, as well as increased knowledge among its participants about research activity. Implementing personalized assent will both respect the child and support biobank research.

## Background

Many biobanks, collections of human biological samples stored for medical-scientific research purposes, include biological samples from children [1–3]. Pediatric biobanking is considered important for generating biomedical knowledge and improving (pediatric) health care [4–9]. Typical for biobank research is that samples can be stored for many years, while linkable to phenotypic data, and that the exact research questions are often not formulated at the time of sample inclusion [10]. Because samples are often stored coded, and not anonymous, and informational risks are involved, ethical (and legal) guidance is needed on appropriate governance, such as consent [11, 12]. Since children are not considered competent and lack the legal capacity to provide informed consent [13], designing an appropriate consent procedure for pediatric biobanking is even more challenging. Generally, parents (or legal guardians) must give permission for the inclusion of their children in biomedical research. Over the last decades, there has been a move towards recognition of a child’s right to be involved in matters that affect him or her and to express personal views [14]. This right is also recognized in biomedical research guidelines, specifically in the requirement to seek a child’s assent. Key guidelines state that when a child is capable to provide assent for participation in biomedical research it should be sought [15–17]. It is considered a necessary, though insufficient condition for the inclusion of children in research [15–17]. This view has also been articulated in the context of pediatric biobank research [18–22]. Earlier we, and others, have argued that assent should be understood from an engagement point of view [23–27]. Assent from an engagement point of view is grounded in respect for the child’s developing autonomy, promoting of or the support for the development of the child and as a support for communication between the researcher and child [23]. To fully acknowledge the differences between children, the assent procedure needs to be adjusted to the individual child. We referred to this as personalized assent [23]. The question follows how the content and process of an assent procedure can be formed in accordance to personalized assent. Moreover, while personalized assent does justice to the individual child, it at the same time creates a dilemma. Accepting a child’s right to personalized assent, as a moral requirement, implies a moral duty of the researcher to make his or her best effort to engage the child. However, this is a positive duty and, without accurate demarcation, a positive duty can be limitless. It is necessary to determine which efforts are reasonable to ask from researchers and how personalized assent can be embedded within the research context. Without further articulation, personalized assent is at risk of becoming an empty concept. In this paper we will therefore provide guidance on howto put personalized assent into pediatric biobanking practice.

## Content of personalized assent

The content of the assent procedure refers to the information that is discussed with the child. Since assent must be understood from an engagement point of view, it follows that the content of the assent procedure is directly linked to the individual child’s capacities and wishes, and therefore varies [23]. For some children it may only be possible to discuss that a blood sample will be taken and that she can say no. For more mature children, the detail of information may be similar to informed consent procedures for adults [26, 27]. Eventually, the child may decide whether she wants to participate in the study as she understands it [28].

### Which information should be discussed first?

There are several empirical studies on children’s understanding of research information and competence to assent/consent [29]. However, it is problematic to combine and generalize the results of empirical assent studies, because of the context specific factors that may have influenced the results [29, 30]. For instance, studies with one simple intervention or type of measurement are easier to understand than studies with a comprehensive research program. Studies on the perception of healthy children found a considerable variation in their understanding of the biobank study rationale [31–33]. It was therefore deemed more appropriate to focus on practical procedures, like a venipuncture [31]. It seems reasonable to focus on concrete information first. Concrete information is easier to understand than abstract concepts, such as privacy issues [34, 35]. Moreover, information about what the short-term experience of the child will be in the study may be most relevant for the child to make up her mind about participation. Biobanking typically consists of three stages: (1) collection and inclusion of the sample, (2) storage of the sample and (3) usage of the sample. The first phase of biobank research entails, for example, a venipuncture or buccal swab. It follows that generally the first phase will be easier to understand and should therefore be the starting point in informing children. The information related to the second and the third stage of biobank research should be offered to children who are able to understand this and moreover, want to receive more information [26]. This would entail topics such as privacy issues and details about the type of studies supported by the biobank.

## The process of personalized assent

The process of the assent procedure refers to how information is offered and the roles of the different persons involved.

### How to offer information?

An important aspect of the way to offer information is the choice of means or material used to inform children. Several means and methods have been suggested to support the transfer of information, such as pictures, games and DVDs (Table 1). Making use of different styles, techniques and technical innovation, can be useful.

**Table 1:** Means and methods to support information transfer

Previously, we and others discussed that combining the classic methods of written information and verbal explanation increases the child’s understanding [23, 70] and that these methods should be used in such a way as to supplement each other [23, 39]. We also suggested the use of other techniques, e.g. pictures. However, merely adding pictures to written information does not seem to increase understanding and additional research is required to optimize communication techniques [71].
One way to improve information provision is the use of stories and/or characters that children are familiar with. This can be helpful in explaining even difficult subjects. Harry Potter or the X-men, for example, can be used to explain genetics and hereditability [72]. Another suggestion is to shape the assent procedure as an activity [58, 73]. This way children truly become part of the research discussion and it seems a promising method to engage them in a way that appeals to them. Examples are creating a storyboard and playing word games as a way to discuss research [58]. Technical innovations can also be used, particularly since present-day children have grown up with multimedia. Although one study showed an increased comprehension of study procedures and risks among children who received multimedia information [74], a review on the improvement of understanding of informed consent elements for adults concluded that multimedia interventions often fail [75]. Moreover, one small study showed that generally children preferred written information sent to them individually, instead of being informed through websites or email [60]. Hence, more research is needed on how to use technological innovations and multimedia effectively. When using multimedia, at least two points need to be considered. First, multimedia can be implemented in a passive form, for example showing a DVD, and/or an active form, for example a computer game. Second, multimedia should not be considered a substitute for interaction between researcher and child [73].

Currently, information technology (IT) interfaces as part of participant-centered initiatives (PCI) are being developed in adult biobanks [36, 37]. These developments can also be used in pediatric biobanking and can play a crucial role in maintaining the relation between the biobank and child (and future adult participant). Interfaces can be used to communicate with children over time and help to address an issue like re-contact. In addition, the quality of biobank research can be improved by investing in ongoing contact with the participating children. Participants are a valuable source for qualitative, experiential, and longitudinal information. An interface can be used to obtain such data [36].

### The role for adults in the assent procedure

Obviously, there must be a contact moment between the child and someone who can assess the child’s capacities and wishes to personalize the assent procedure. Typically, there is a triangular relationship between the child, the parent(s) and the researcher [38].

The researcher is an obvious candidate to seek assent from the child. The researcher is the one who wants to study the child (or the biological material) and is probably most knowledgeable about the research. However, one of the main dilemmas of assigning the role to obtain personalized assent to the researcher is his or her personal interest in including the child in the study. It may be appealing not to invest much effort in informing the child and elaborating on the child’s opinion. Selecting research staff who is aware of their responsibility to respect the child and have a sense for working with children is therefore indispensable. In addition, training the persons who will obtain assent is very important. They should know what the aim of assent is, how to offer information to children, how to assess their capacities and wishes and how to act on it [39, 40]. Parents know their child and may have particular insights into interpreting verbal and non-verbal signals. Therefore, they can have an important part in the assent procedure. They can advise or assist in explaining (parts of) the study. In addition, parents may also play an important role in protecting their child’s right to dissent and/or assent if a researcher does not take his or her responsibility to protect these rights [41]. However, it must not be overlooked that parents can also disrespect their child’s dissent [31, 41, 42] and can be opposed to their child’s right to assent or consent [43, 44].

### The assessment itself

The next question is how a researcher (or research staff) can assess the individual child in order to personalize assent. The first thing that obviously needs to be considered is whether communication with the child is possible. When communication is possible, first the basic research information should be offered. Hereafter, the researcher needs to find out what the child understands, what the child wants to know and what the child can and wants to decide [45]. Factors that are considered to influence these matters for example are, psychological state, anxiety, level and types of (research) experience, health status, maturity, culture, religion, familial and societal context and complexity of the research [29, 30, 45–50]. During a personal conversation the researcher should attempt to find out what the child understands by asking to explain their understanding of the study in their own words [46]. In addition, researchers need to listen and respond to the concerns and questions of the child. The aim of this conversation is for researchers to ascertain what information is valuable to this particular child and to try to fit the information to the child’s needs [45]. Some may say that it is difficult for researchers to assess children. However, this does not mean that it should not be strived for [46, 51]. Moreover, some consider assessing a child’s capacities not as difficult as it may seem [52].

### Subjectivity of the assessment

A dilemma with the above approach is that it relies heavily on the researcher’s capacities and efforts to optimize the assent procedure. If a researcher fails to invest in the assent procedure sufficiently, there is a risk of not involving the child at all, or at least not enough. As discussed, this risk originates from the researcher’s interests in including the child (or her biological material) in the research. Therefore, they may not be fully committed to optimizing the assent procedure. This may be particularly the case where researchers view assent as an extra and parental consent as the only legal or ethical requirement. Furthermore, presumptions of the researcher about the incapacities of children may lead to failure to include children in the research discussion [53]. Introducing a presumption of competence for children as a starting point in the research discussion about biobank participation has been suggested as a way to ensure that children are taken seriously [54]. Further studies of such an approach and the attitudes of the persons who seek assent will be valuable. In addition, well-validated tools to assess a child’s capacities to assent/consent can be helpful to objectify the assessment outcome [55, 56]. However, since the aim of assent is to engage with children, we think there remains a central role for the researcher in the assent procedure.

### The reaction of the child

It is reasonably straightforward that an affirmative agreement of the child constitutes assent and that a clear objection refers to dissent. How should we consider the grey area between a clear dissent and an affirmative agreement? It is quite possible that children stay silent when their parents have given permission for participation and they feel intimidated [28, 57]. Although children are aware of the possibility to dissent, they find it difficult to say no in reality [32, 33]. Furthermore, when children do not give a clear answer about whether they want to participate, it is questionable whether they have understood the research information [58]. Thus, especially when children keep silent, extreme caution must be exercised before proceeding with the study.

## Discussion

Having described the practical considerations for the implementation of personalized assent, several comments can be made. First, the question arises whether assent is a strict requirement in pediatric research. An important distinction here is assent understood as a procedure and assent understood as the affirmative agreement of the child. The assent procedure is itself formative; appropriate engagement adds to the integrity of the research relationship and its meaning for both researcher and child. Only when it is absolutely impossible to follow the assent procedure, the duty of the personalized assent procedure may be waived by a research ethics committee (REC) beforehand, for example in research with newborns. Otherwise a researcher must make the effort to seek assent in practice. Whether an affirmative answer of the child is required, however, is more complicated. It is possible that during the assent procedure, it becomes clear that the child does not have the capacities or desire to be involved in the decision-making procedure [26, 59]. Since we conclude that not all children can and/or want to give assent after going through the assent procedure, it would be strange to impose an affirmative agreement of the child as a strict requirement on researchers. Therefore, we argue that provided that other safeguards are in place, i.e. the requirement of parental permission, strict regulations about acceptable risks, supervision of a REC, the responsibility of researchers to respect their participants and respect for dissent [23], a child may be included in a study without her affirmative agreement. However, this caveat should not be used as a way to circumvent the general duty to make the effort to seek assent. In addition, when there is no affirmative agreement of the child, the onus of proof is on the researcher to show that he or she did honor the personalized assent procedure and that it is justified to continue the research. Note that when the research population consists of children who are quite mature and/or the proposed study is reasonably straightforward, it will be more difficult to prove that it is justified to continue with the study without an affirmative agreement.

Second, investing in a personalized assent procedure can serve two goals. An appropriate involvement of the child shows respect for the child as a person. It is the moral (and professional) responsibility of the researcher to put effort into the engagement of the child in the research discussion and personalized assent [23]. It is interesting to see that children themselves also articulate some sort of duty on the part of the biobank researcher to involve them in the research discussion based on reciprocity: when they participate, researchers have a responsibility to treat them with respect [60]. It is important for researchers to be aware of this expectation and trust: it is another reason to take seriously their responsibility to engage children. Next to respect for the child, an appropriate involvement of the child in the research discussion also contributes to the quality and success of the research [41, 60]. It may lead to a general trust in the biobank and children who are well informed and intrinsically motivated to participate, will probably provide more accurate information and are likely to participate longer [30, 41, 60–62]. This is especially important for biobanks, since they generally conduct longitudinal research and often want to collect phenotypic information on a regular basis. Earlier we argued in favor of re-contacting former child participants when they become adults, in order to give them the opportunity to withdraw their samples [63]. When participants can identify themselves with the biobank goals, they are probably less likely to withdraw their samples.

Third, in the introduction we described that the duty to involve children in the research discussion is a positive duty and needs further demarcation. The amount of effort required to invest in the child must be reasonable [64]. There will be practical limits to this responsibility, for example the development of interactive games may be too expensive for small biobanks. Moreover, the efforts required must be proportionate to the study, hence, to the characteristics of the biobank. For example, taking a one-time saliva sample for a few measurements differs considerably from monthly blood withdrawal and providing information by filling in several questionnaires. There is also a difference between biobanks that want to conduct simple tests, like a hemoglobin measurement, compared to biobanks that want to use DNA sequencing methods. In general it can be stated that the greater the burdens and/or higher the risks, the greater the effort that must be put into the assent procedure.

Fourth, in line with the former remark, ethics governance can play both a formative and controlling role. RECs can explain what the underlying aim of assent is and advise on how to put it into practice. In addition, they can request a thorough assent policy as part of a research or biobank proposal in order to create a system of checks and balances [20]. This policy must clearly describe the entire assent procedure, including data on the persons who will be obtaining assent and which information materials will be used. The proposal should also discuss the course of action when the child does not give an affirmative reaction. A local REC can check whether the proposed assent policy is appropriate and proportionate for that particular biobank.

Fifth, one could argue against personalized assent that it would be impractical and/or not enforceable [65, 66]. As discussed previously, we are aware that personalized assent is an appeal to a researcher’s integrity and that it is difficult to provide fixed end points [23, 67]. The assent procedure must be flexible enough to adapt to different (biobank) studies and the different situations of individual children, for example, their difference in maturation, diversity in family dynamics or culture [26, 30, 68]. Researchers must be reminded of their professional responsibility and moral duty to engage children and strive for empowerment of the individual child [46]. Since biobank employees reported a natural desire to engage children in the consent procedure out of respect for the child [41], we are confident that an appropriate awareness can be achieved. However, making this duty explicit and increasing awareness of researchers and biobank employees is needed.

Last, we want to remark that not only assent, but also consent procedures can benefit from working from the engagement point of view. Although informed consent nowadays gained an important legal function, our view emphasizes the ethical origins of consent. People differ in informational interests and in the ways they want to be informed [69]. Viewing consent more from an engagement point of view may add to honoring these differences.

## Conclusions

Personalized assent is aimed at engaging children in accordance to their personal capacities and desires. The assent procedure can be designed in order to support the goal of engaging children. Particularly for biobanks, the characteristic three phases, i.e. inclusion, storage and use of samples, provide a natural arrangement of the information. Since issues related to the first phase are usually most concrete and relevant for the child, it is sensible to start with information about the first phase. Topics linked to the other two stages could be added according to the child’s desires and capacities.

Investing in the assent procedure as such should be a requirement for pediatric research and (biobank) researchers must invest in the assent procedure. However, since some children do not have the capacity or desire to be involved in the research discussion, an affirmative agreement of the child cannot be a strict requirement. It is important to note that researchers should still strive for such an agreement, and that the onus of proof is on the researcher to justify continuing research without an affirmative agreement of the child by showing that they made a proportionate investment in a personalized assent procedure.

Although personalized assent will require certain efforts, the pediatric (biobank) community must be aware of its importance. The investment and trust earned, if maintained, can result in ongoing engagement, important longitudinal information, and stability in/for the research infrastructure, as well as increased knowledge among its participants about research activity. Implementing personalized assent will both respect the child and support biobank research.
